# Supplementary material for: Solvent Free Three-Component Synthesis of 2,4,5-trisubstituted-1H-pyrrol-3-ol-type Compounds from L-tryptophan: DFT-B3LYP Calculations for the Reaction Mechanism and 3H-pyrrol-3-one↔1H-pyrrol-3-ol Tautomeric Equilibrium
Source: Molecules. 2020 Sep 25;25(19):4402. doi: 10.3390/molecules25194402 (PMC7582317; doi:10.3390/molecules25194402)
Supplement: Supplementary file 1 [file molecules-25-04402-s001.pdf]

Supplementary Information

Solvent Free Three-Component Synthesis of 2,4,5-trisubstituted-1*H*-pyrrol-3-ol-type Compounds from *L*-tryptophan: DFT-B3LYP Calculations for the Reaction Mechanism and 3*H*-pyrrol-3-one↔1*H*-pyrrol-3-ol Tautomeric Equilibrium

Diego Quiroga *, Lili Dahiana Becerra and Ericsson Coy-Barrera

Bioorganic Chemistry Laboratory, Facultad de Ciencias Básicas y Aplicadas, Universidad Militar Nueva Granada, Campus Nueva Granada, Cajicá 250247, Colombia; lilidahiana18@gmail.com (L.D.B.); ericsson.coy@unimilitar.edu.co (E.C.-B.)

* Correspondence: diego.quiroga@unimilitar.edu.co

Content

1. Figure S1: ^{1}H NMR spectrum of **1a** in $CDCl_3$.
2. Figure S2: ^{13}C NMR spectrum of **1a** in $CDCl_3$.
3. Figure S3. HR-MS spectrum of compound **1a**.
4. Figure S4: ^{1}H NMR spectrum of **1b** in $CDCl_3$.
5. Figure S5: ^{13}C NMR spectrum of **1b** in $CDCl_3$.
6. Figure S6. HR-MS spectrum of compound **1b**.
7. Figure S7: ^{1}H NMR spectrum of **1c** in $CDCl_3$.
8. Figure S8: ^{13}C NMR spectrum of **1c** in $CDCl_3$.
9. Figure S9: ^{1}H NMR spectrum of **1d** in $CDCl_3$.
10. Figure S10: ^{13}C NMR spectrum of **1d** in $CDCl_3$.
11. Figure S11. HR-MS spectrum of compound **1d**.

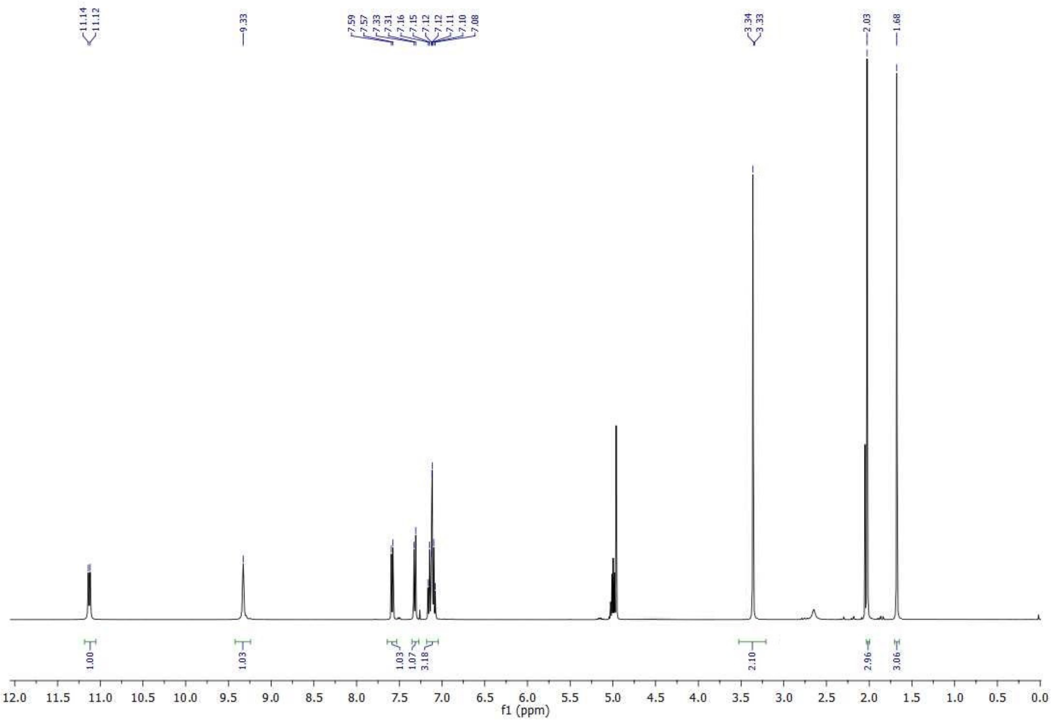

Figure 1. ^1H NMR spectrum of **1a** in $CDCl_3$.

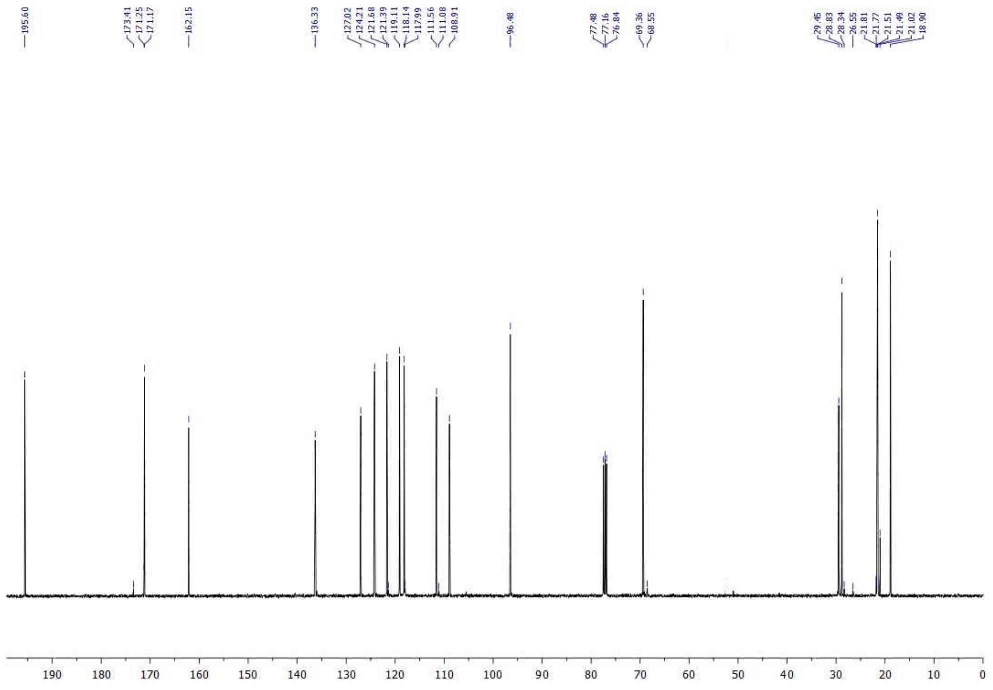

Figure 2. ^{13}C NMR spectrum of **1a** in $CDCl_3$.

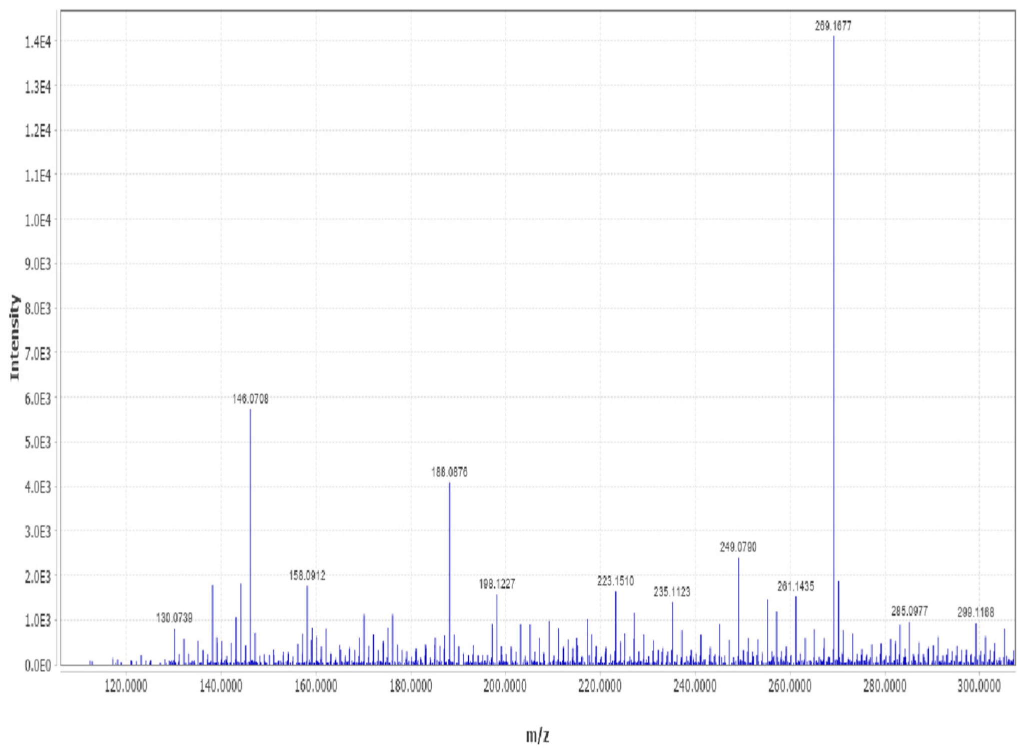

Figure 3. HR-MS spectrum of compound **1a**.

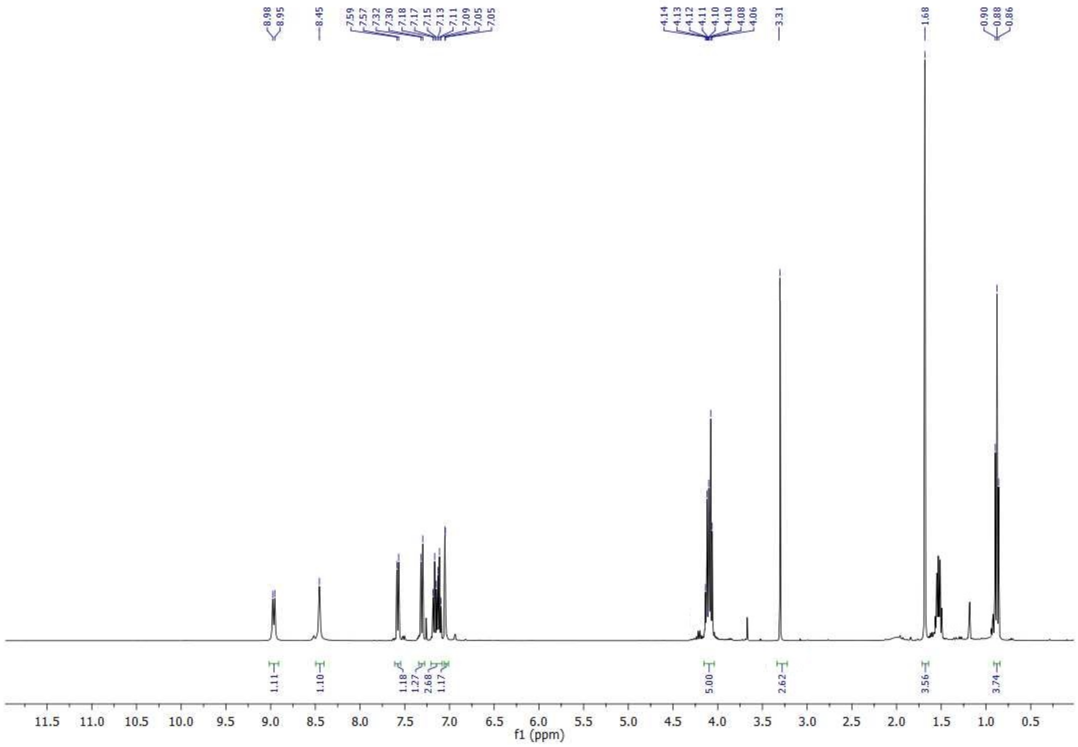

Figure 4. ^1H NMR spectrum of **1b** in $CDCl_3$.

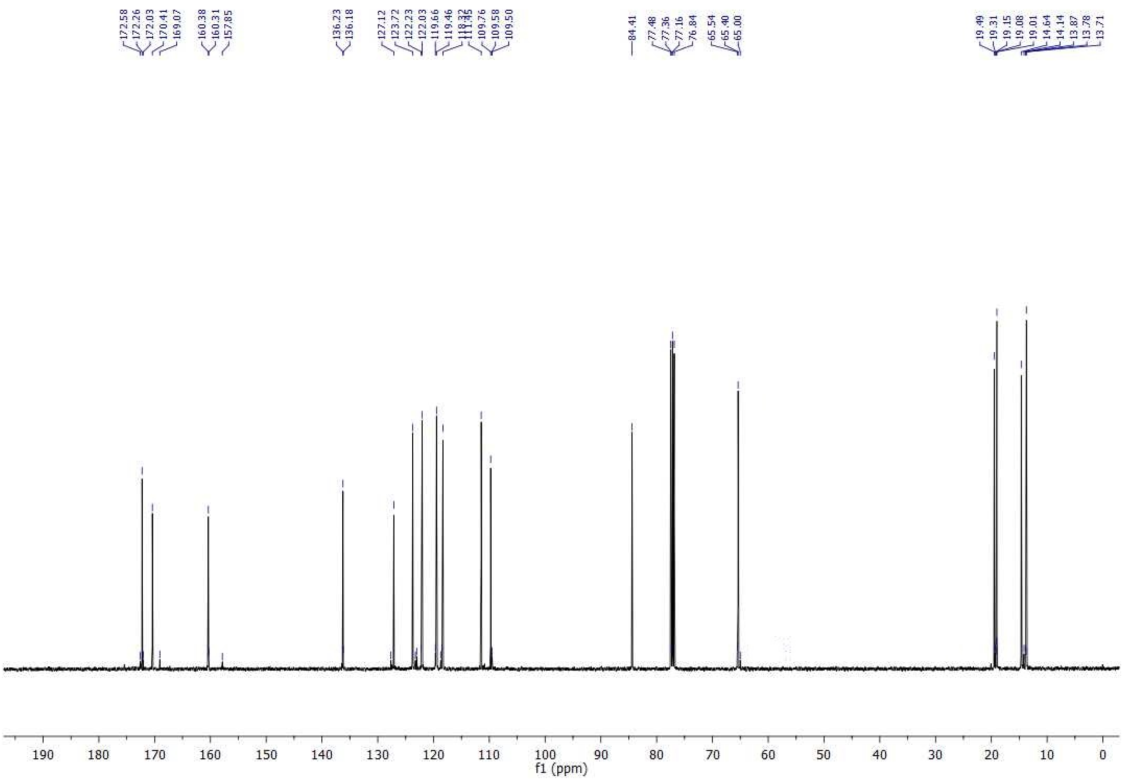

Figure 5. ^{13}C NMR spectrum of **1b** in $CDCl_3$.

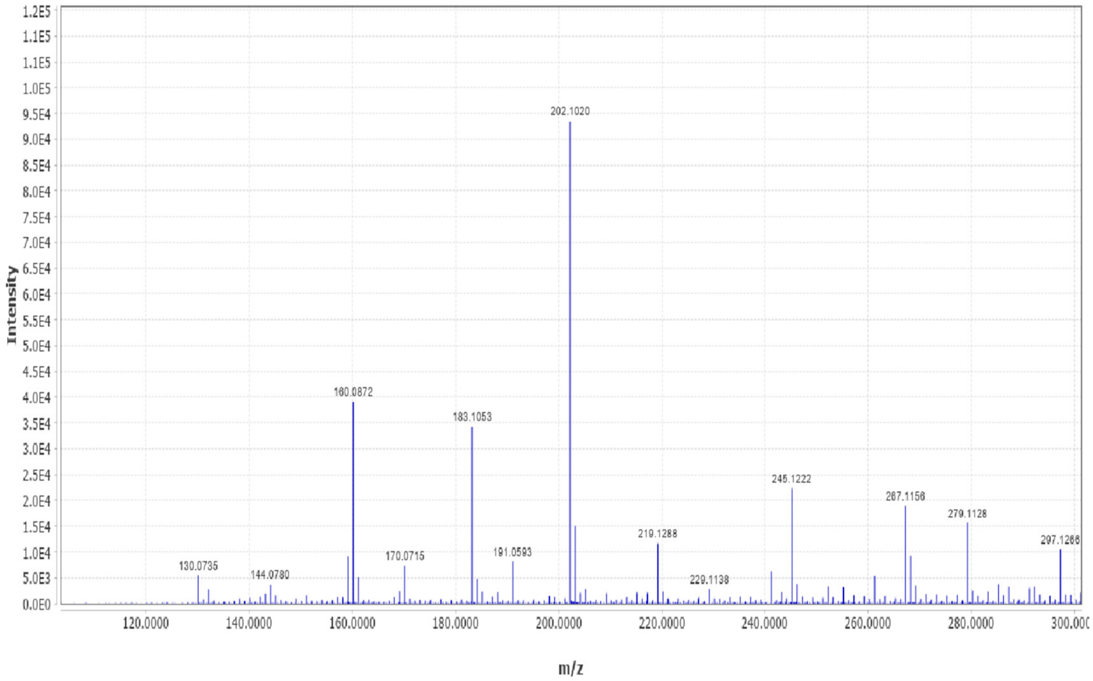

Figure 6. HR-MS spectrum of compound **1b**.

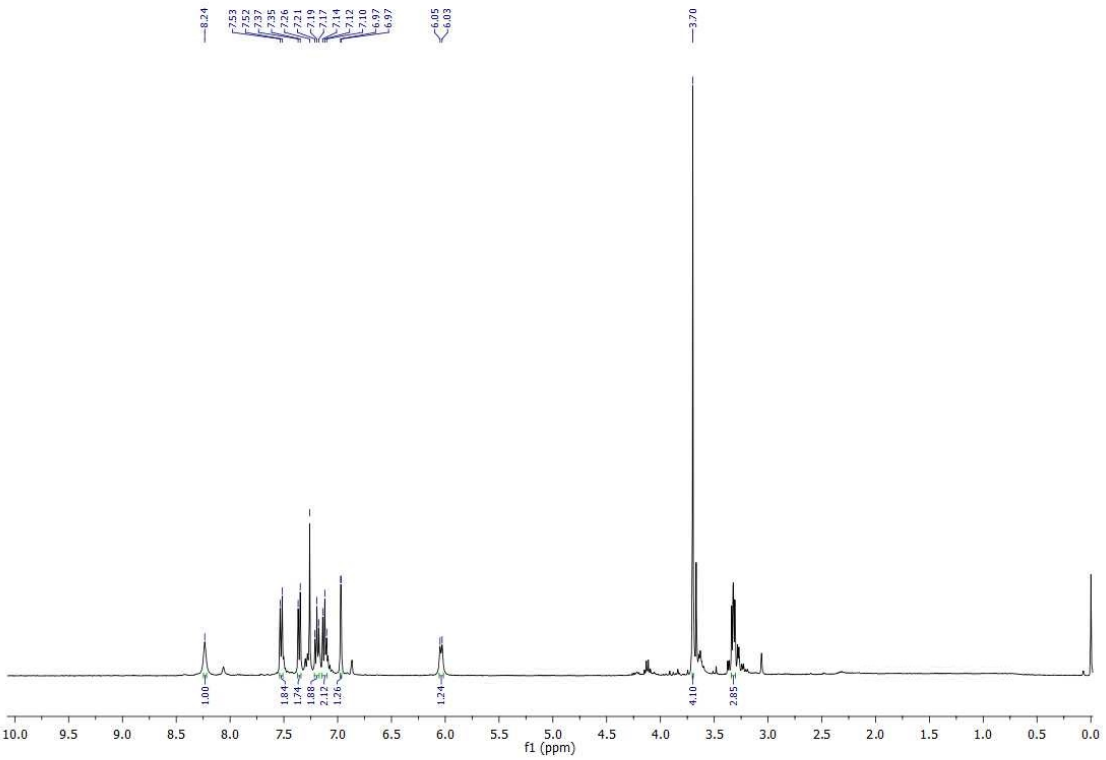

Figure 7. ^{1}H NMR spectrum of **1c** in $CDCl_3$.

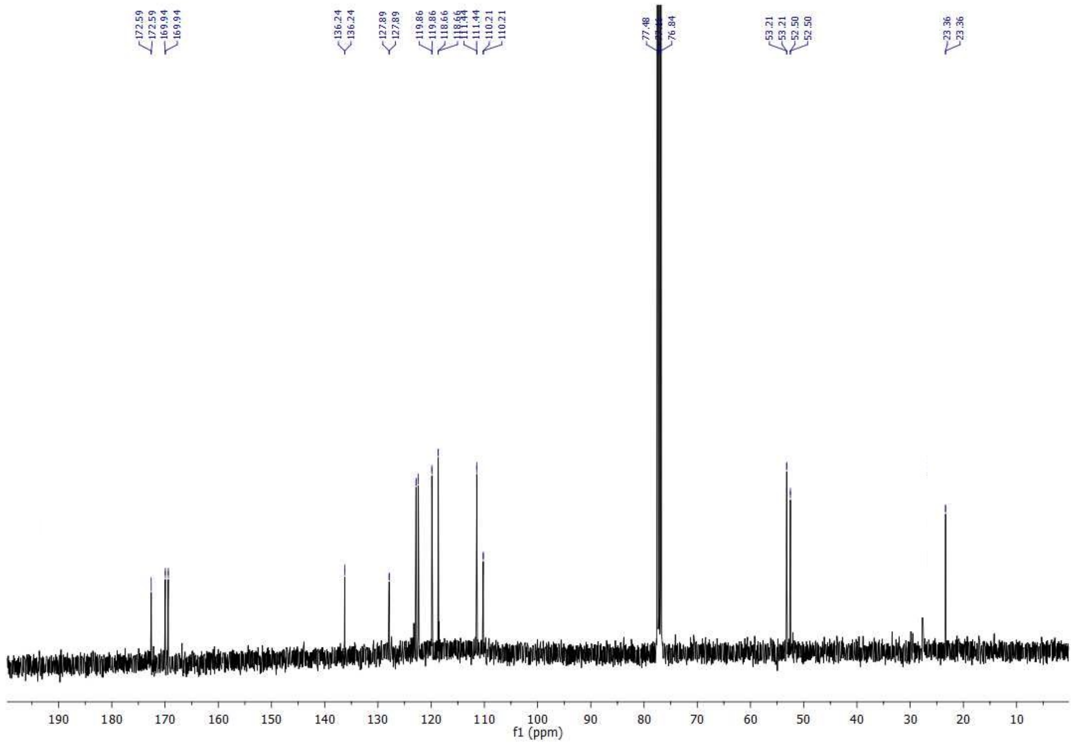

Figure 8. ^{13}C NMR spectrum of **1c** in $CDCl_3$.

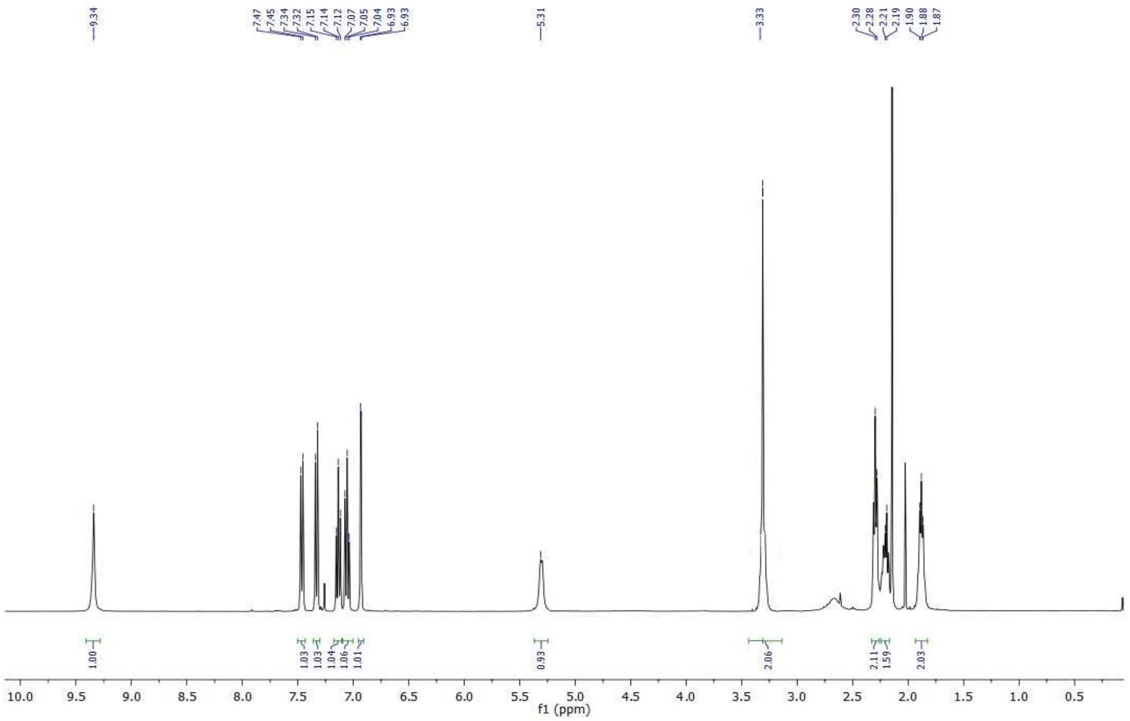

Figure 9. ^{1}H NMR spectrum of **1d** in $CDCl_3$.

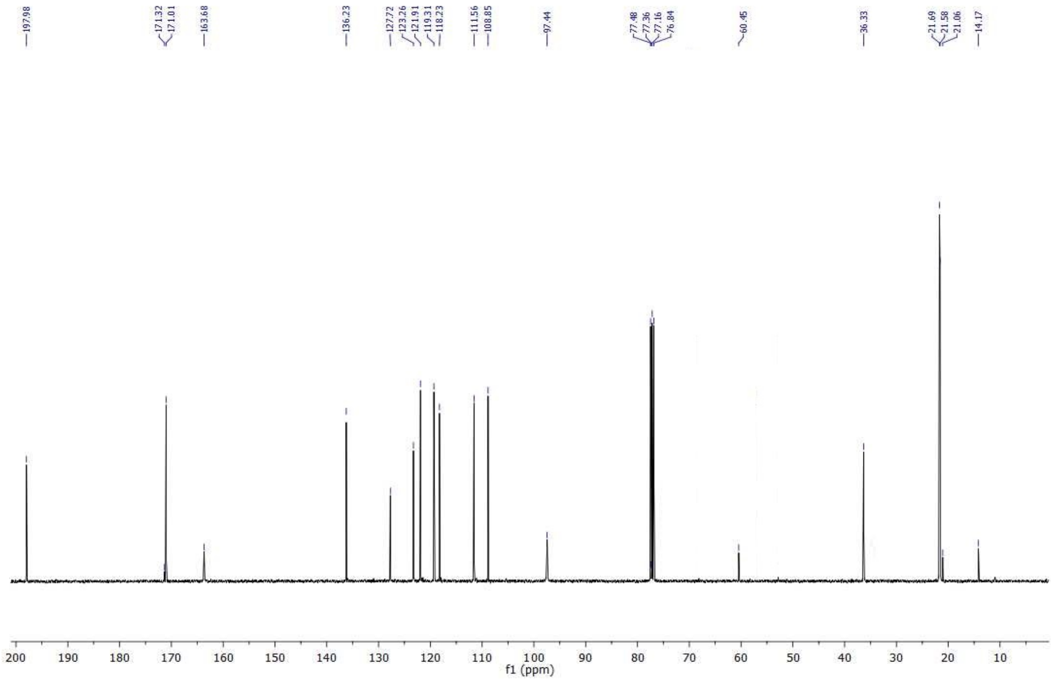

Figure 10. ^{13}C NMR spectrum of **1d** in $CDCl_3$.

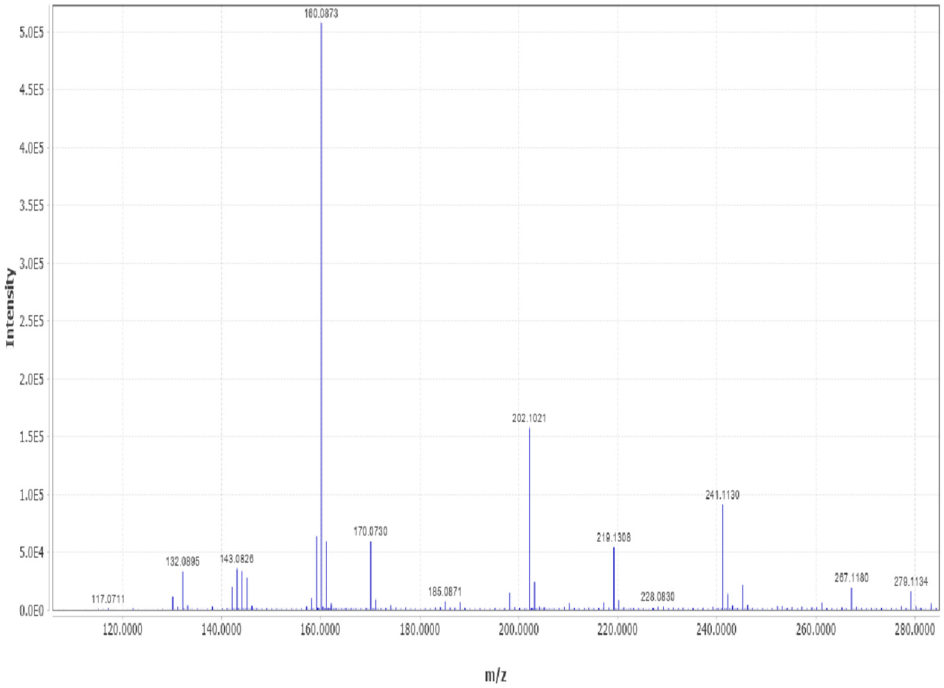

Figure 11. HR-MS spectrum of compound **1d**.
